# Increasing Fatigue Life of 09Mn2Si Steel by Helical Rolling: Theoretical–Experimental Study on Governing Role of Grain Boundaries

**DOI:** 10.3390/ma13204531

**Published:** 2020-10-13

**Authors:** Sergey Panin, Ilya Vlasov, Pavel Maksimov, Dmitry Moiseenko, Pavlo Maruschak, Alexander Yakovlev, Siegfried Schmauder, Filippo Berto

**Affiliations:** 1 Institute of Strength Physics and Materials Sciences SB RAS, 634055 Tomsk, Russia; vlasov.ilya.viktorovich@gmail.com (I.V.); mpv@ispms.ru (P.M.); moisey@rocketmail.com (D.M.); 2 School of Advanced Manufacturing Technologies, Tomsk Polytechnic University, 634055 Tomsk, Russia; alexandryakovl@gmail.com; 3 Institute for Materials Testing, Materials Science and Strength of Materials (IMWF), University of Stuttgart, 70569 Stuttgart, Germany; siegfried.schmauder@imwf.uni-stuttgart.de; 4 Department of Industrial Automation, Ternopil Ivan Puluj National Technical University, 46001 Ternopil, Ukraine; maruschak.tu.edu@gmail.com; 5 TomskNIPIneft JSC, 634027 Tomsk, Russia; 6 Department of Mechanical and Industrial Engineering Faculty of Engineering, Norwegian University of Science and Technology, NO-7491 Trondheim, Norway; filippo.berto@ntnu.no

**Keywords:** helical rolling, heterogeneous materials, grain size, fatigue, cracking, cellular automata, grain boundary

## Abstract

The structure and mechanical properties of the 09Mn2Si high-strength low-alloyed steel after the five-stage helical rolling (HR) were studied. It was revealed that the fine-grained structure had been formed in the surface layer ≈ 1 mm deep as a result of severe plastic strains. In the lower layers, the “lamellar” structure had been formed, which consisted of thin elongated ferrite grains oriented in the HR direction. It was shown that the five-stage HR resulted in the increase in the steel fatigue life by more than 3.5 times under cyclic tension. The highest values of the number of cycles before failure were obtained for the samples cut from the bar core. It was demonstrated that the degree of the elastic energy dissipation in the steel samples under loading directly depended on the area of the grain boundaries as well as on the grain shapes. The fine-grained structure possessed the maximum value of the average torsional energy among all the studied samples, which caused the local material structure transformation and the decrease in the elastic energy level. This improved the crack resistance under the cyclic mechanical loading. The effect of the accumulation of the rotational strain modes at the grain boundaries was discovered, which caused the local structure transformation at the boundary zones. In the fine-grained structure, the formation of grain conglomerates was observed, which increased the values of the specific modulus of the moment of force. This could be mutually compensated due to the small sizes of grains. At the same time, the coarse-grained structures were characterized by the presence of the small number of grains with a high level of the moments of forces at their boundaries. They could result in trans-crystalline cracking.

## 1. Introduction

In recent years, the beginning of a new era in the development of materials science associated with “heterogeneous materials” has been actively discussed in many publications [1]. In fact, heterogeneous materials are postulated as “the next hot area of research after the nanomaterial era”. Their definition is that, “Heterogeneous materials are materials with a high degree of heterogeneity of the strength properties from one domain to another.” This can be the result of (i) the diversity of the microstructure, (ii) heterogeneity of the crystal structure, or (iii) inhomogeneity of the chemical composition. The sizes of structurally inhomogeneous domains can range from micro- to millimeters, while their shapes can vary significantly.

The mechanism of a multiple increase in the mechanical properties of heterogeneous materials is explained by the different strains in soft (plastic) and hard (high-strength) domains [1]. This provides (i) the possibility of accommodation of strains in neighboring areas of different strengths and (ii) the formation of strain gradients due to the development of counter stresses at grain boundaries and dislocation pile-ups, which do not cause micro-discontinuities. At the same time, the complementary action of two mechanisms is discussed, i.e., back-stress-induced strengthening and strain hardening due to the sufficiently large sizes and spacing of the domains [2].

Obviously, most structural materials are heterogeneous by definition, ranging from widely used low-carbon steels of the ferrite–pearlite class, moving to duplex and MAX-phase steels, and ending with laminate composites of alternating hard and soft layers. Nowadays, the “hierarchical steels” term is gaining special popularity in some papers [3]. The active use of the transmission electron microscopy and nanoindentation methods enables approaching the solution of the problem of designing high-strength materials (steels) with increased ductility (plasticity) from the standpoint of the targeted formation of nanostructured precipitates, grain boundaries, and subgrains, as well as substantiation of the special role of dislocations [4]. Additionally, the effect of heterogeneity of the (nano)structure has been also discussed, in particular, due to the formation of a nanolaminate structure of austenite and martensite microlayers.

In [5], the effect of microstructural features of the SA508 Grade 3 Class I low-alloy steel (LAS) has been investigated. The propagation of short cracks below the ΔK_th_ threshold value, as well as limitation of the movement of dislocations in front of the crack tip and, accordingly, slowing its velocity when approaching the grain boundaries, have been found experimentally. The non-uniform mechanism of the propagation of short cracks does not enable applying all known macro-dependences for predicting their behavior, in particular the Paris equation. This once again emphasizes the demand for studying these patterns for fatigue cracks at the micro-, meso-, and macro levels. One of the successful examples of this approach is the results published in [6], which has proposed methods for assessing the fatigue life of resistance spot welded steel joints in the zones of shear stresses under cyclic tension. New engineering algorithms for calculating stress intensity factors and J-integral using three-dimensional finite element analysis have been successfully combined with fractographic studies of the fracture surfaces.

In the light of the above-mentioned publications, the priority directions in the field of designing structural materials should be considered: (i) the formation of laminate phases (at the levels from macro- to nano), the difference in properties of which enables improving both strength and ductility (as well as fracture toughness at the crack propagation stage); (ii) the formation of a composite structure at the nanoscale level, also assuming the presence of ductile and hard phases; and (iii) a multilevel hierarchical organization of heterogeneous structures that make it possible to absorb input energy by their self-consistent changes at all structural levels without any “critical” discontinuities [7].

Nevertheless, these priorities in the aspects of forming the structure of the materials possess significant limitations. According to [4], if grain refinement is the only mechanism to improve strength, this is accompanied by a decrease in ductility, which corresponds to the typical representation in the framework of the Hall–Petch law. This effect becomes critical for grain sizes below 1 µm, because the ductility of the materials decreases sharply in this case.

When discussing the key factors affecting the ductility of layered composites, the following terms are used: thicknesses of layers, the structure and adhesion of interfaces, gradients of property changes, the strain-hardening coefficient, strain rate, branching of cracks upon reaching the interface, blunting of crack tip, crack overlapping, redistribution of stresses, local strain hardening, interphase separation, etc. [7]. It should be noted that the strain processes should be divided into two groups: preceding and accompanying failures. In addition, it should be considered that the role of interfaces in these two process types can substantially vary.

The idea of designing hierarchical materials has been developed in [8]. In an effort to form a composite with a metal matrix and a multiscale hierarchical structure by powder metallurgy, a Ti- (TiBw/Ti) one has been synthesized. It contains the (i) layered, (ii) lattice, and (iii) needle-like structures. This has enabled achieving the effect of hardening, reinforcement, and plasticization. As a result, the inverse relationship between strength and ductility, described in publication [9], has been overcome.

Completing the analysis of current approaches for designing the heterogeneous materials, functionally graded ones (composites) should be noted [10]. Initially, the functionally graded materials, such as Ti-TiB, have been designed to prevent cracking upon high-temperature processing due to different thermal expansion coefficients. In this case, thermal stresses can be effectively reduced, as well as the plastic strain development and the metal phase decomposition can be delayed by the load redistribution on ceramic inclusions [11]. It has been shown that main cracks begin to branch near the interface between layers, which prevents catastrophic fracture. This effect provides good ballistic properties of such materials that enable using them for the protection against impact loads.

Various methods to form gradient structures have been discussed in some publications. For example, the surface modification (for instance, by concentrated flows of charged particles) has been widely applied to increase fatigue life [12,13,14]. However, according to the authors, the approaches based on the initiation of shear strains under the highly constraint conditions are of the greatest interest. In particular, high-pressure torsion (HPT), for example, is an important area of research into the fragmentation and mass transfer processes in crystalline materials under the extremely constrained strain development. This enables avoiding cracking when reaching high strain degrees [15]. Consequently, small samples can be processed, including austenite/ferrite duplex stainless steels. The strain development heterogeneity along the diameter of a disk has been shown. A double-swirl pattern has emerged on the top surface at the early HPT stages. These two centers of the swirl have moved toward the disc center as the numbers of HPT revolutions has been increased, and ultimately, the double-swirl has evolved into a single one. It has been shown in a similar paper dedicated to the processing of high-purity aluminum disks [16] that the initially inhomogeneous microhardness distribution over the cross-section levels has aligned with an increase in the number of HPT revolutions, and “results are interpreted using a model in which the degree of hardness depends upon the rate of recovery in the material”.

Of particular interest is helical rolling (HR). Many papers devoted to the HR processing of various types of materials have been published to date. For example, the HR of the 40Cr steel bar (analog to AISI-5140) steel has been analyzed in [17]. It has been shown that the microstructure of the peripheral region has possessed mainly equiaxed sub-ultrafine grains with a size of about 5 μm. The bar core has had a characteristic banded (as in a conventional “rolled” one) texture of narrow grains elongated in the rolling direction with dimensions of (5–10) × (0.9–1.5) µm^2^, and chromium carbide chains with individual crystals of 200 nm or less. Interpretation of the obtained results, as a rule, has been proposed in terms of the development of the severe plastic strain processes [18,19].

The same method, but referred to as “three-roll planetary milling” (PSW), has been used to modify the structure of copper rods in [20]. The PSW process has effectively reduced grain sizes from about 28 µm down to less than 1 μm in the near-surface region while maintaining the coarse-grained inner surface of the rod with grain sizes from a few to tens of microns after six PSW passes. Another study of the effect of the PSW process on the structure and properties of pure magnesium has been carried out in [21]. In this case, the PSW has been done in several passes with a gradual decrease in temperature (down to room one). After six passes, yield strength has reached 1.116 MPa, and elongation has been 13% with a high degree of homogeneity of the structure over the billet.

During the PSW process, a number of its parameters are varied. In [22], using an example of copper pipes, the effect of temperature (above the recrystallization one) has been studied, which has been associated with the heat energy generated by severe deformation and friction work. The processes of dynamic recrystallization and grain growth have been analyzed within the framework of numerical simulations by the finite element method. The possibility of a significant increase in microhardness by such processing has been shown. The results of using the “radial shear rolling” method for processing titanium rods have been presented in [23]. The relationships between the stress–strain state parameters, hardness, and grain sizes have been assessed by numerical experiments. In general, the HR method is positioned in publications as a way of inner architecting by means of severe plastic strains. It is postulated that severe grain recovery, controlled by the self-organization of the structure, is realized through fast mass transfer, the formation of new phases, and various “reaction–diffusion” works [24].

The results of investigations of the HR of commercially pure titanium are given in [25]. It has been shown that the formation of a submicrocrystalline state has been accompanied by the presence of highly dispersed carbide particles. This has caused an increase in yield strength, tensile strength, Young’s modulus, and fatigue life during tension under the high-temperature creep conditions. Additionally, the thermal stability of high-strength SMC titanium has been improved. From the other hand, the formation of nanoporosity upon the HR process has decreased fatigue limit at room temperature. When presetting the optimal HR modes for processing titanium and its alloys, the controlled factors have been the type of the formed microstructure and the possibility of its modification. In [26], the transformation of the initial lamellar microstructure of the Ti-6Al-4V alloy into the globular one has been analyzed. The HR process at a temperature of 940 °C and a reduction ratio of 1.25 has been described using finite element simulations in terms of the stress–strain state evolution.

The results of research on the HR processing of silumin have been presented in [27]. It has been shown that an alternating strain pattern during the HR has provided a reduction in grain sizes by a factor of 5-6 compared with that of the longitudinal rolling. Computer simulation of the radial-shear rolling has been carried out using the finite element method [28]. The analysis of the simulation data has been done in terms of variation in the rigidity coefficient values under stress condition along the billet radius near the end, which has enabled the authors to give recommendations on choosing the optimal rolling mode.

In the papers of Academician V.E. Panin, the structural modification of the Fe-Cr-Mn metastable stainless austenitic steel has been described [29]. It has been realized by the “warm” HR processing followed by “cold” rolling in conventional rolls. As a result, lattice curvature areas have been formed. On the basis of the obtained results, the authors have concluded that the optimal lattice curvature value enables reducing the cold brittleness threshold and improving the steel fatigue life. Scratch tests have revealed a strong increase in the damping effect in the formed hierarchical nanocrystalline grain structure, which has been accompanied by the generation of hexagonal close-packed (hcp) ε-martensite in hcp grains and body centered cubic (bcc) α’-martensite. The high damping level has been explained by the high concentration of nanoscale mesoscopic structural states arising in the crystal lattice curvature areas upon the high-temperature HR processing. These effects have been due to the self-consistent mechanical behavior of hcp ε-martensite lamellas in hcp austenite grains and bcc α’-martensite arising upon cold rolling of the steel after the high-temperature HR [30].

Finally, one of the key features of the HR method is the formation of gradient structures, in which both the sizes of structural elements and their strength properties have been changed. Some publications have discussed the effect of the strength gradient formation on an increase in resistance to fatigue fracture [31]. Using the 304 austenitic steel as an example, a promising strategy of the formation of the gradient structures to improve fatigue resistance has been demonstrated through the formation of both positive and negative strength gradients in the sample. In this case, the specified gradient has been provided not by the microstructure modification but by the loading conditions.

It should be concluded based on the foregoing that the formation of the gradient structures by the HR is a very effective way to improve the mechanical properties and to minimize the possibility of critical discontinuities. Last but not least, this method is relatively easy to implement technically. This also enables maintaining a high ductility level. In addition, the graded structure is able to provide enhanced fatigue life.

The purpose of these studies has been to establish patterns of the modification of the micro- and macrostructure of the 09Mn2Si high-strength low-alloyed (HSLA) steel along the cross-section of the round bar (rod) for the multi-stage HR and to analyze the effect of this modification on the resistance to strains and fracture under static and cyclic loading. The steel is widely used both in the construction of pipeline systems as well as for manufacturing structural elements for various applications. In order to find the regularities of the influence of the grain structure parameters on the behavior of the material with different sizes and shapes of grains under cyclic loading, a series of numerical experiments have been carried out by the stochastic excitable cellular automata (SECA) method [32,33].

## 2. Materials and Methods

The high-temperature multi-stage HR of the 09Mn2Si steel (Table 1) was carried out using an “RSP 14-40” (ISPMS SB RAS, Tomsk, Russia) three-roll mini helical rolling mill with a capacity of 33 kN in a temperature range of 1123–773 K (850–500 °C) with a gradual decrease in temperature at each subsequent pass. Cold water quenching was performed after each rolling pass. The layout of the rolls in the HR mill was previously described in [34], where this processing was referred to as “three-roll skew rolling”. In this case, a bar was preheated in a furnace to a predetermined temperature, after which the HR process and subsequent quenching were carried out. Then, the degree of reduction of the bar and the heating (processing) temperature were changed. Thus, the HR process was repeated until the required degree of the bar strains was reached. The initial bar diameter was 40 mm, and it was 18 mm after the HR. The total degree of the true logarithmic strain of the steel was 0.8 after five passes (the relative strain was 73.93%).

**Figure 1:**
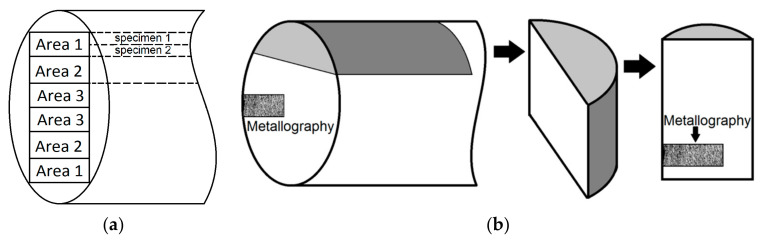
(**a**) A scheme of the bar and the places where the samples were cut out; (**b**) schematic representation of the areas where the metallographic analysis was carried out: (**left**) the cross-section view, (**right**) a view along the rolling direction.

After the HR, the rod macrostructure was gradient (the maximum strain corresponded to the outer layer, while it was minimal in the core). For this reason, the bar was conditionally divided into three areas, which are indicated in Figure 1 by the digits 1, 2, and 3. Samples for testing were fabricated using plates cut from each of the three indicated areas in an amount of at least four from each one (Figure 1).

The “dog-bone” shaped samples for mechanical testing were cut from the bar by electrical discharge machining. The gauge cross-section size was 16 × 5 × 1 mm^3^. Static tensile tests were performed using an “Instron 5582” (Instron, Norwood, MA, USA) electromechanical testing machine with a load rate of 0.6 mm/min.

In addition, the same samples were tested under cyclic tension using a “Biss UTM 150” (Bangalore Integrated System Solutions, Bangalore, Karnataka, India) servo-hydraulic testing machine with a cycle asymmetry of *R* = 0.1, at a maximum load of 270 MPa and a frequency of 10 Hz. During the tests, the sample surface images were taken by a “Canon 700D” (Canon Inc., Tokyo, Japan) camera under static tension with an interval of 10 s and by a “Basler piA 2400-17gc 5M” (Basler AG, Ahrensburg, Germany) video camera under cyclic tension with an interval of 1000 cycles. Strain fields were calculated using the “Vic-2D” software (Version 2, Correlated Solutions, Inc, Irmo, SC, USA), which is an experimental strain calculation system implemented the digital image correlation method. The method is based on a non-contact (optical) procedure for assessing displacements with their subsequent recalculation into strain fields. Such calculations used a mesh of square-shaped elements, the size of which was selected in order to ensure the minimum error in the obtained results.

An additional transverse notch with a depth of 0.4 mm was made on the samples for the cyclic tension tests from one of the thinned part edges to localize the fracture processes and to enable a more detailed observation of the fatigue crack propagation.

The crack opening values were calculated, and elongation was measured using a virtual extensometer and the “Vic-2D” software (Correlated Solutions) [35]. For this purpose, the virtual extensometer marks were placed on optical images along the fatigue crack tips. The parameters were calculated using data on the displacements of these marks and embedded routines.

The microstructure was studied by a “Carl Zeiss Axiovert 25” optical microscope (Carl Zeiss, Oberkochen, Germany). The samples were etched with a 5% nitric acid solution. Ferrite grain sizes were determined according to ASTM E 112-96. Microhardness values were measured using a “PMT-3” device (LOMO, St-Petersburg, Russia) (the load on the Vickers pyramid was 0.98 N). After mechanical testing, lateral faces of the fractured samples and their fracture surfaces were examined using a “LEO EVO 50” scanning electron microscope (Carl Zeiss, Oberkochen, Germany).

## 3. Results and Discussion

### 3.1. Metallography

**Figure 2:**
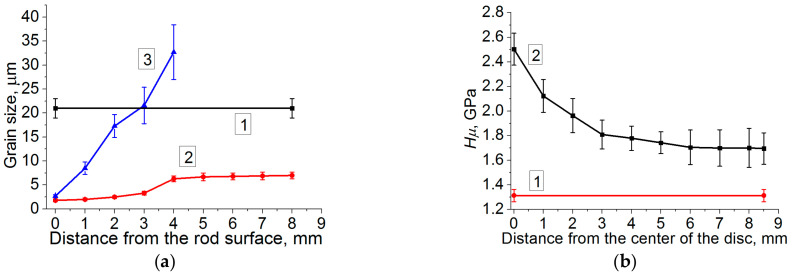
(**a**) Grain sizes vs. the distance from the bar surface: 1—as-received; 2—across the helical rolling (HR) direction; 3—along the HR direction; (**b**) microhardness values: (1)—as-received; 2—the bar cross-section.

**Figure 3:**
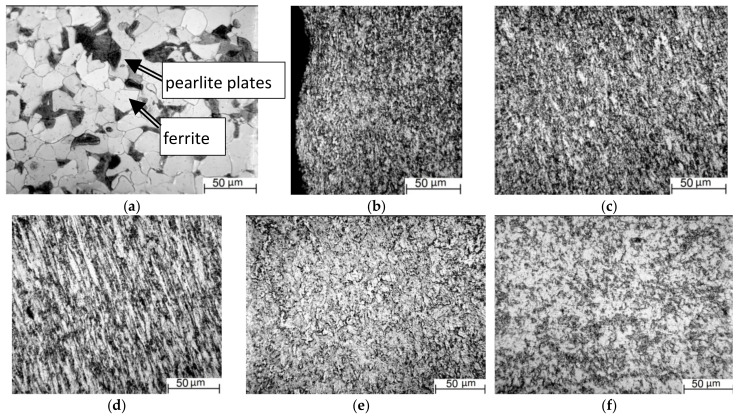
Optical images of the 09Mn2Si steel microstructure: (**a**)—as-received; after the HR: along Table 1; (**b**) at the bar edge; (**c**) at a distance of 1.5 mm; (**d**) at a distance of 4.0 mm; across the rolling direction; (**e**) at the distance of 4.0 mm; (**f**) the bar core.

The as-received 09Mn2Si steel possessed the ferrite–pearlite structure with a slight striping and characteristic grain sizes of 21 ± 2 μm (Figure 2a, curve 1). At a higher magnification, cementite laths were distinguished in individual pearlite grains (Figure 3a).

After the five-step HR processing, the most intense “stretching” of grains was in the rolling direction (Figure 2a), while only a decrease in their sizes relative to the initial state was across. Thus, a “lamellar” structure had been formed in the core (Figure 3d), which consisted of thin elongated ferrite grains oriented in the rolling direction. A finely dispersed structure had been formed in a surface layer about 1 mm deep that had been subjected to the maximum strains upon the HR process. It possessed no pronounced texture traces (Figure 3b).

Optical microscopy data obtained in the surface layer of the sample after the HR showed a fine structure that consisted of ferrite and fractured pearlite plates (Figure 3b,e). In the longitudinal section at a depth of more than 1 mm, individual ferrite grains were distinguished (Figure 3c). They were preserved after strains and elongated in the longitudinal direction with a slight deviation toward the bar core. This deviation was caused by the bar strains not only in the rolling direction, but also toward the core as a result of a decrease in its size and the preset orientation of the rolls. In the transverse direction, such grains appeared similar to equiaxed light inclusions, which were sometimes slightly elongated (Figure 3e,f).

Moving to the bar core was accompanied by an increase in the sizes and numbers of distinguishable elongated ferrite grains. Their rolling texture was clearly visible at a distance of 4 mm from the surface (Figure 3d). At this depth, ferrite and pearlite grains were found in the transverse direction. As a result of strains during the HR, pearlite interlayers between ferrite grains were barely distinguishable. Boundaries of structural elements looked “torn” due to their mixing (at the meso-level according to the “shear + rotation” scheme) upon the processing. As was shown in [36] by an example of intense strains of a triple junction of grains, this gave rise to the development of rotational wave-like mass transfer flows along the grain boundaries. According to the authors, the result of the action of these flows appeared in the “corrugation” or “raggedness” of the grain boundaries.

### 3.2. Static Tension

**Figure 4:**
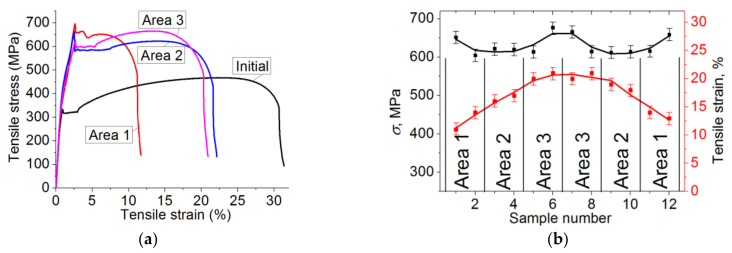
The stress–strain diagram (**a**) and the graph of the mechanical properties (**b**) for the samples cut from different bar areas.

Figure 4a shows stress–strain diagrams obtained during the static tensile tests of the samples cut from different bar areas. The dynamics of changes in the values of ultimate strength and elongation are shown in Figure 4b. Quantitative data on the key mechanical properties of the samples are summarized in Table 2. 

In the tensile diagram of the as-received steel, there were (i) both the upper and lower yield points, (ii) the long parabolic hardening stage, and (iii) high values with respect to elongation (typical for low-carbon steels, Figure 4a. After the HR, there was a significant increase in tensile strength, both the upper and lower yield points, while elongation was reduced. The highest values of the deforming stresses were characteristic of the samples cut from the bar edge (area 1) due to the finest grain inside.

When moving to the bar core and increasing average grain sizes (area 2), the strength characteristics gradually decreased, which was accompanied by an increase in the elongation level (Figure 4, Table 2). The obtained data confirmed the gradient pattern of the macrostructure formed during the HR.

For the samples cut from the bar core (area 3), both high values of ultimate strength (above 600 MPa) and ductility (elongation of more than 20%) were characteristic. According to the authors, this effect was associated with the formation of the textured (in the rolling direction) and, at the same time, fine-grained (across) structure.

The obtained results indicated that the HR processing, even with the significant decrease in the grain sizes, enabled preserving the microscale plastic flow mechanisms, which reduced the risk of strain localization and retained a high relaxation ability (in contrast to the equal-channel angular pressing (ECAP) method). However, the formed large grain boundaries prevented the development of plastic strains and increased the material strength properties.

**Figure 5:**
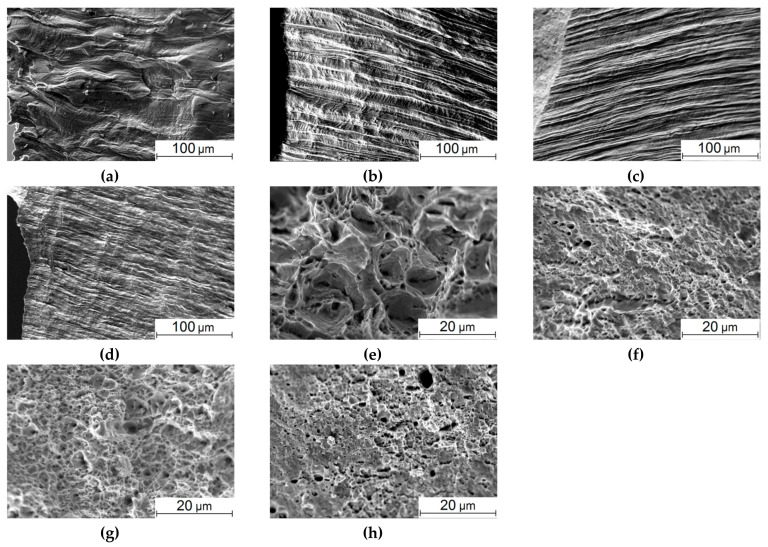
SEM micrographs of the flat surfaces (**a**–**d**) and the fracture ones (**e**–**h**) of the 09Mn2Si steel samples failed under static tension: as-received (**a**,**e**) and after the HR (**b**–**d**,**f**–**h**): area 3 (**b**,**f**); area 2 (**c**,**g**), area 1 (**d**,**h**).

SEM micrographs of flat surfaces and fracture ones of the samples that failed under static tension are shown in Figure 5. On the flat surfaces, grain reliefs appeared as a result of strains during the tests, reflecting the internal structure (Figure 5a–d). For the as-received steel, deformed ferritic grains elongated along the tensile axis, and sliding traces were visible (Figure 5a). After the HR, the surfaces had a longitudinal texture consistent with highly elongated ferritic grains (Figure 5b–d). At the same time, sliding traces were only in the samples from the bar core (Figure 5d). With increasing distance from the core, the width of the longitudinal folds on the surface reduced (Figure 5c,d), which was in good agreement with the decrease in the grain sizes revealed above. On the surface of the samples from the near-surface area, small transverse microcracks were visible that indicated less ductility (Figure 5d).

The fracture surface of the as-received steel sample had a dimpled pattern typical of ductile low-carbon ones (Figure 5e). After the HR, the sizes of characteristic dimples were significantly reduced (Figure 5f–h), which correlated with the decrease in both the grain sizes and ductility. In addition to the dimple relief, pores and individual microcracks were found on the fracture surface of the sample from area 1 (Figure 5h).

The cracks in the samples from area 1 were evenly located in the thinned cross-section (neck). The authors failed to identify individual areas with higher or lower crack density. The authors attributed this homogeneous distribution of cracks to a limited sample volume, within which there was no pronounced gradient of properties or changes in the sizes and shapes of structural elements. The formation of cracks was only observed in the samples from area 1. The authors suggested that the reason for this fact was the high material hardness in this zone and, as a consequence, higher brittleness (in comparison with other zones 2 and 3).

### 3.3. The Fatigue Life Tests

The fatigue life tests of the 09Mn2Si steel were carried out for both the as-received samples and the ones after the HR. The scheme of their manufacturing fully corresponded to that for the static tests (Figure 1). It was found that fatigue life increased by at least 2.5 times as a result of the five-stage HR (Table 3). For the samples from area 1, where the highest microhardness values were observed, durability increased up to 200,000 cycles. For the bar core samples, the number of cycles to failure increased by a factor of 3.8 over that of the as-received state. Table 3 presents data on the number of cycles at the main crack nucleation (*N_i_*) and propagation (*N_p_*) stages, as well as the relative duration of the nucleation stage (*N_i_/N_f_*), which is further used in discussing the causes of the observed phenomena.

**Figure 6:**
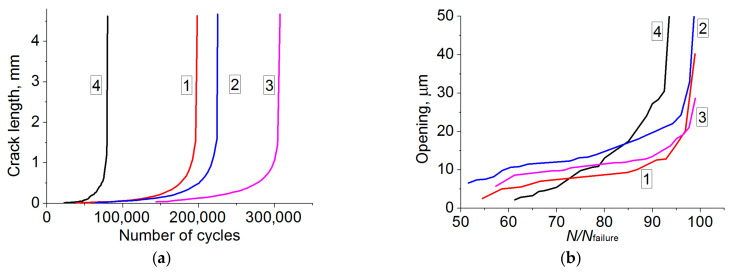

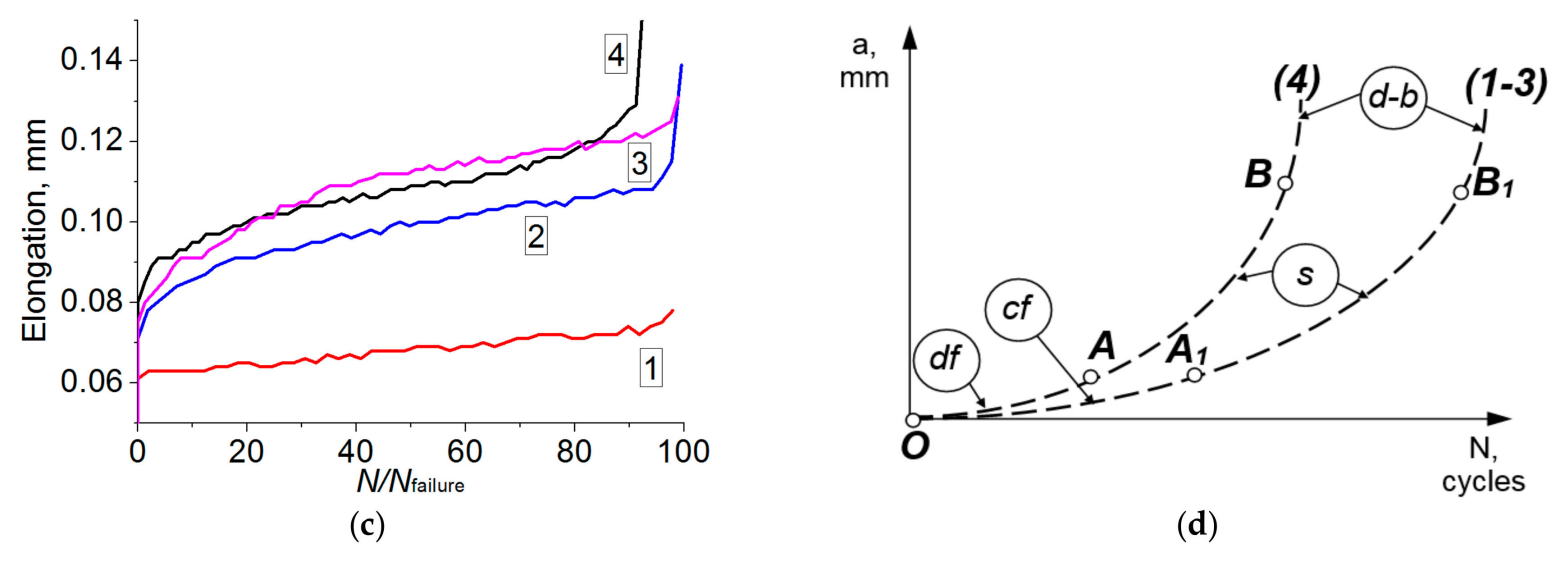
The diagrams of the fatigue fracture parameters the 09Mn2Si steel samples after the HR: (**a**) the crack length as a function of the number of loading cycles; (**b**) opening (according to the VIC-2D terminology); (**c**) elongation (according to the VIC-2D terminology); area 1 (1); area 2 (2); area 3 (3); as-received state (4); (**d**) the physical and mechanical mechanisms of the crack propagation for the samples (1–3) and (4): OA (OA_1_)—the crack nucleation; AB (A_1_B_1_)—the crack propagation; after B (B_1_)—the quasi-static final fracture; df—the straight front; cf—the crack front branching; s—fatigue striations; d–f—the ductile–brittle final fracture.

**Figure 7:**
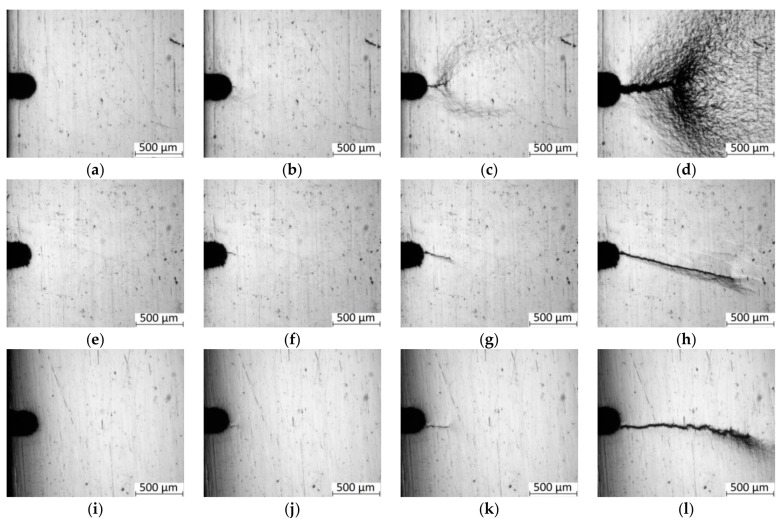
The photographs of the sample surfaces after the cyclic tension tests: (**a**–**d**) as-received; (**e**–**h**) after the HR (area 1); (**i**–**l**) after the HR (area 3). (**a**) 0 cycles; (**b**) 40 × 10^3^ cycles; (**c**) 60 × 10^3^ cycles; (**d**) 75 × 10^3^ cycles; (**e**) 0 cycles; (**f**) 100 × 10^3^ cycles; (**g**) 150 × 10^3^ cycles; (**h**) 190 × 10^3^ cycles; (**i**) 0 cycles; (**j**) 180 × 10^3^ cycles; (**k**) 240 × 10^3^ cycles; (**l**) 300 × 10^3^ cycles.

Using digital photographs of the sample surfaces upon testing, graphs of the cyclic durability (dependence of the crack length on the number of loading cycles) were drawn (Figure 6a), and the magnitudes of the main crack opening were shown as well (Figure 6b). The starting point (the beginning of the crack propagation stage) was considered at the moment when the crack length reached ≈80 μm (it became visible, Figure 6b). For the as-received steel sample, a higher crack opening rate was found throughout the observed range *N/N_f_* = 60–100%. This was consistent with a higher material ductility (Figure 5e and Figure 7d), as well as a higher fracture toughness of the sample due to the larger grain sizes. In the samples after the HR, a significant increase in the crack opening rate was at the final fracture stage (*N/N_f_* > 90%). In general, the magnitude of the main crack opening changed in a similar way for the samples cut from areas 1–3 (Figure 6b, curves 1, 2, and 3).

The “elongation” graphs for the samples (with a crack) could be interpreted as the value of their elastic–plastic compliance upon loading cycles (in Figure 6c, it was plotted in the normalized coordinates of the number of loading cycles). The lowest elongation was characteristic of the sample from area 1, which had to be associated with the highest resistance to cyclic deformation in fatigue crack tip. This value was approximately one and a half times higher for the samples cut from area 2 (curve 3).

Finally, the “elongation” values were maximum and approximately close for the both as-received steel and cut from area 3, as well as the slopes of the corresponding dependences (curves 3 and 4). This result was consistent with the higher toughness of these materials, despite the fact that the plastic zones at the crack tips looked very different (Figure 7d,l).

The curve shapes were typical in terms of the cycle life diagram (Figure 6a). Significantly lower fracture toughness showed the as-received steel samples, while the curves were shifted to the right for the samples cut from areas 1–3. This indicated their greater cyclic durability, which was mainly due to the stage of macro-defect nucleation. At the same time, the curves changed in a similar way despite a noticeable difference in the structure and some variance in the number of cycles before failure. This result fully corresponded to the typical material fatigue resistance concept. It is known that the initiation and the beginning of the propagation of short cracks was a structure-sensitive process, because the dimensions of defects and plastic zones at their tips were comparable with the sizes of grains [37,38]. The fatigue crack propagation was a structurally insensitive process if the defect dimensions significantly exceeded the grain sizes [39,40], which was observed in this case. These prerequisites were fully consistent with the obtained result and the data in Table 3, since the number of cycles for the samples cut from all three areas was close at the crack propagation stage *N_p_*, =157 × 10^3^ ÷ 180 × 10^3^. This indicated that the fatigue crack was similar for the samples cut from each of the three areas, while the resistance to accumulation of scattered damages (fatigue stage 1) was higher in the samples with the pronounced laminate texture (area 3).

Next, the photographs of the sample surfaces (Figure 7), which were obtained upon the process of cyclic loading, were analyzed. For the as-received steel samples (Figure 7a–d), the *N_i_/N_f_* ratio was 30% (Table 3). Since the tests were carried out at high stresses close to the yield point, there was a slight shrinkage of the material near the notch at the crack initiation stage (Figure 7b). Then, a pronounced plastic strain zone was formed in front of the crack as two localized shear bands (Figure 7c). As the crack propagated, its size increased and was decorated with a strain relief [37]. The crack branched during its propagation, and plastic strain was most pronounced especially at the final stages (Figure 7d).

The samples cut from area 1 possessed the lowest *N_i_/N_f_* = 20%, i.e., the crack had nucleated early enough, but it propagated for a long period of time (Table 3). Upon the main fatigue crack nucleation (Figure 7f), there were no signs of tightening; the crack propagated with minimal traces of plastic strain without branching (Figure 7g). At the final stage, there were lines of steps next to the main crack parallel to the direction of its propagation (Figure 7h). This was accompanied by a slight sample tightening. However, in comparison with the as-received steel sample, there was a significantly more brittle fracture pattern, and plastic strains were weakly expressed at the final stage (this is shown in the corresponding fractographic imagers below.

The maximum ratio *N_i_/N_f_* = 45% was typical for the samples cut from area 3. There was no plastic tightening at the crack nucleation stage (Figure 7j); the crack branched during its propagation, and slight plastic strains were discernible at its tip (Figure 7k). At the final stage of the fatigue crack propagation, there was a weak tightening (Figure 7l). Thus, the samples cut from area 3 possessed an optimal set of the properties: the maximum duration of the crack initiation stage and the fatigue crack resistance values at the level of the samples cut from area 1 with the maximum strength characteristics and the minimum grain sizes.

These patterns were summarized in Figure 6d, which enabled emphasizing the importance of the sizes of structural elements (grains) at the short crack nucleation and (partly) propagation stages (OA and OA1 Sections). Although the mechanisms of the long fatigue crack propagation and then sample failures had significant individual morphological variations, it could be interpreted as the same type in general. This fact was confirmed by the fractographic analysis of SEM micrographs.

Figure 7 shows the curvatures of the “long crack” fronts. Previously [41], the authors used a special method to analyze the effect of plastic strains at the tip of both short and long cracks on the macro- and micro-mechanisms of their propagation. Considering those results, it could be assumed that the curvature of the long crack (Figure 7) was associated with a change in the stress–strain state at its tip. Increasing the crack length caused the localization of strains, enhancing the crack tip opening and its blunting upon individual loading cycles. During the next loading cycle after the crack tip blunting, not only pull-off stresses presented at this point, but also shear strains arose. This caused a change in the trajectory of the crack propagation. In the studied case, these effects were observed precisely for long cracks, which coincided with the results published in [41].

**Figure 8:**
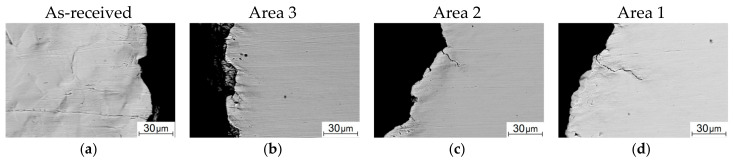

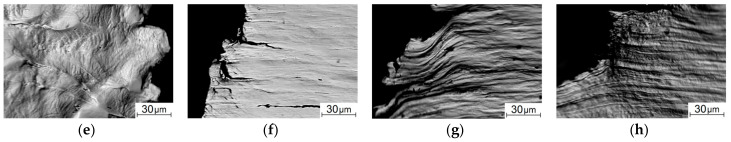
The SEM micrographs of the flat sample surfaces: as-received (**a**,**b**) and after the five-stage HR (**c**–**h**); (**a**,**c**,**e**,**g**)—the stable crack propagation zone; (**b**,**d**,**f**,**h**)—the necking zone (pre-fracture).

The SEM micrographs of the flat surfaces of the as-received samples and ones after the five-stage HR are shown in Figure 8. The flat surface of the as-received sample was characterized by a weakly pronounced strain relief in the stable fatigue crack propagation zone (Figure 8a). It should be noted that the crack propagation front was fairly uniform; only individual traces of its branching were found. With further main fatigue crack propagation, plastic strains caused striped dislocation slips in ferrite grains (Figure 8e). After the five-stage HR, the crack front had signs of branching in its stable propagation zone (Figure 8b–d). Moreover, it was not perpendicular to the load direction in some areas. This could activate both pull-off and shear stresses at the crack tip, and it also contributed to an increase in the inhomogeneity of the stress–strain state of the material and its significant changes in individual sections of the crack front [42]. The strain-induced relief had been formed mainly near the main crack propagation zone. In some places (the samples cut from areas 1 and 2), there were individual brittle microcracks, which were also a consequence of the macro-discontinuity branching upon its propagation (Figure 8c,d). The pre-fracture area possessed a pronounced banded relief corresponded to the lamellar texture of the steel (Figure 8f–h). In general, the strain-induced reliefs observed on the surfaces correlated with the patterns shown in Figure 5b–d.

**Figure 9:**
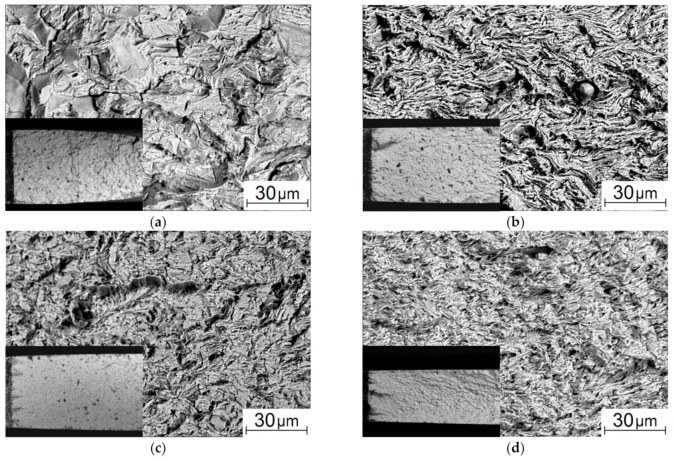
The SEM micrographs of the sample fracture surfaces: as-received (**a**) and after the five-stage HR (**b**–**d**); the stable crack propagation zone. (**a**) as-received; (**b**) area 3; (**c**) area 2; (**d**) area 1.

**Figure 10:**
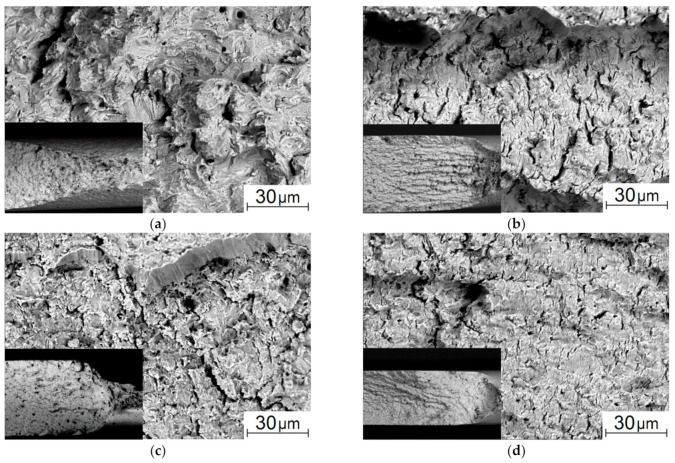
SEM micrographs of the sample fracture surfaces: as-received (**a**) and after the five-stage HR (**b**–**d**); the necking zone (pre-fracture). (**a**) as-received; (**b**) area 3; (**c**) area 2; (**d**) area 1.

After the cyclic tensile test, the fracture surface of the as-received steel sample was represented by the trans-crystalline fracture of the stable crack propagation zone (Figure 9a). In the necking (pre-fracture) zone, the fracture surface had a ductile–brittle appearance, because fatigue fracture developed at the load values close to the yield point (Figure 10a), and significant plastic strains had already had a dominant effect on the crack propagation mechanisms [43,44]. After the five-stage HR, the trans-crystalline fracture with a ridge relief was in the stable crack propagation zone. However, dimensions of its fragments were significantly smaller than those in the as-received steel. It could be assumed [3,45] that this was due to the structure formed during the HR (Figure 9b–d). Variations between the samples cut at the bar edge and from its core were that the longitudinal texture was more strongly traced in area 3 (Figure 9b), while small elements of the fractographic relief had a more equiaxed shape in area 1 (Figure 9d).

Here, it was also necessary to consider that one of the mechanisms of the formation of such a structure had been the nucleation of microrotation chains in the grain bodies, which subsequently had caused their fragmentation [36]. As a result, the total area of the interfaces had increased. Moreover, as shown in [36], the rotation chains in the grain bodies had been formed as half-loops with their ends closed at the boundaries. This kind of fragmentation had given rise to a very indented relief of the interface (which had been energetically extremely disadvantageous in the case of an equilibrium crystal, but had taken place upon the HR process).

However, the pattern of the fractographic relief noticeably changed in the necking zone, which, among other things, included a noticeable number of microcracks (Figure 10b–d). This indicated significant localized plastic strains [4,45]. In particular, longitudinal grooves oriented parallel to the surface were in the sample cut from area 3. In the samples cut from both areas 2 and 1, (i) there were fewer microcracks, (ii) there were practically no such longitudinal grooves, and (iii) the elements of the fractographic relief were quite small (Figure 10c,d). Note that the number of loading cycles was comparable for the samples after the HR cut from different areas: *N_p_*, =157 × 10^3^ ÷ 180 × 10^3^; it varied insignificantly at the fatigue crack propagation stage.

Since the key distinctions in the fatigue life of the studied samples were at the crack nucleation stage, corresponding computational experiments were carried out. The effect of the grain structure type on the strain behavior precisely at the stage before the macro-discontinuity (macrocrack) nucleation was studied.

### 3.4. Simulation of the Behavior of Structures with Different Grain Sizes and Shapes under Cyclic Mechanical Load Conditions

In order to describe the intense high-rate deformation processes, the authors developed the original SECA method described in detail in [32,33,36,46]. It should be noted that this approach was proposed specifically for simulating processes in open systems with continuous power supply. It is known that the processes of self-organization and the nucleation of new structures were possible precisely in such systems. For the description of thermodynamically quasi-equilibrium quasi-static processes, there were typical approaches well-proven and experimentally tested many times. Nevertheless, as previously shown by the authors, the results obtained by the SECA method were in good agreement with the theory of elasticity.

A significant difference between the proposed approach and most discrete methods (for example, molecular dynamics or mobile cellular automata) was the fact that an active element described not a discrete mass of material, but a fixed space region where material fluxes developed. That was, in terms of finite volume methods [47], the SECA element modeled the “control volume”, while the finite element methods in mechanics operated on the representative volume. This concept opened up broad prospects for the numerical simulation of matter and energy flows of a most diverse nature.

Within the framework of this method, each element of the space volume was modeled in the form of an active element, the state of which qualitatively characterized the local response of the medium and in a certain way depended on the entropy value. The parameters of each cellular automaton at each time step of the algorithm were determined using the theory of fuzzy sets. A simulated sample filled the space area and was divided into elementary volumes that were modeled by a cellular automaton. In addition to the state, the automaton was characterized by the parameters corresponding to the simulated volume of the medium. An excitable automaton was able to undergo a sequential set of switching from one state to another under external exposure (energy fluxes). Each automaton element was characterized by a certain set of neighbors on the first three coordination spheres, as well as numerical parameters corresponding to the material contained in the simulated space volume (elastic modulus, density, shear modulus, dislocation density, specific thermal conductivity, specific heat, thermal expansion coefficient, etc.).

In addition, explicit consideration of the polycrystalline structure of the material, i.e., the presence of internal interfaces in it, was realized. The network of active elements that made up the sample was divided into clusters. Each cluster modeled a grain characterized by its own orientation of the crystal lattice with given Euler angles. Based on them, the angle of misorientation at the interface was determined for adjacent grains. It was one of the important parameters that affected the boundary energy characteristics. The developed algorithm for modeling the transfer of mechanical energy was supplemented with blocks for calculating the vectors of the moments of forces acted on the local mesovolume.

Since the description of the core of the computational algorithm was published quite well (for example, [32,33,36,46]), the authors considered it expedient to focus on the specifics of the description of the processes most relevant for the materials obtained by the HR process. Due to the fact that the HR had formed materials with the structure in which rotational and shear strain modes played the dominant role, the method was developed for the case of explicit consideration of intergranular slip processes based on the Mott model [48] for the atomic level.

**Figure 11:**
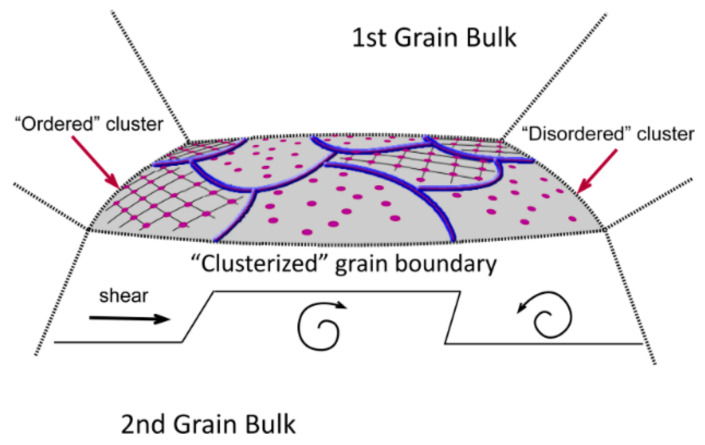
A schematic representation of the “clustered” grain boundary with the crystal lattice curvature.

So, if two crystallographic planes were in contact with each other but had different orientations, it was explicitly considered that the contact zone was a planar subsystem with a cluster structure within the framework of physical mesomechanics [36]. These “areas” were separated by zones with increased curvature of crystal lattices (“disordered clusters”). At the mesoscale, the lattice curvature in such zones could be expressed in terms of the elastic torsional energy [33,36,46]. As noted in [49], “Grain boundary bulges within or crossing the mantle, will be subjected to a non-uniform shear, and will rotate with increasing strain, leading to the asymmetric shapes.” At the same time, there were many clusters of adjacent grains, the lattices of which were well overlapped on top of each other. This is shown schematically in Figure 11.

At the microlevel, the elastic torsional energy arose as a result of a set of elementary atom displacement acts in the perpendicular and tangential directions relative to the interface. This was expressed in the formation of an inhomogeneous field of normal and shear stresses. The sliding speed of the grain boundaries in the material (*v*) was determined by the following relation:

(1) v=Aσ e−BRT

where *σ* was the grain boundary stress; and *A*, *B*, and *R* were coefficients. It should be noted that this relationship was similar to the modified Turnbull equation for describing material flows within the SECA method.

Next, two identically oriented crystallographic planes in a single crystal with a hexagonal close packing were considered, and the required sliding activation energy was determined, where *h* was the distance between planes. The stress *σ* required for a small displacement *x* was determined as follows:

(2) $$\sigma =\frac{Gx}{h}$$

where *G* was the shear modulus in a given direction. For large displacements:

(3) $$\sigma =\frac{Ga}{2\pi h}\sin\frac{2\pi x}{a}$$

where *a* was the distance to the nearest point of stable equilibrium (interatomic distance):

(4) $$a=\frac{2h}{\sqrt{3}}$$

The activation energy *U* for displacement of the plane by half the interatomic distance was calculated in the following form:

(5) $$U=\Omega \int_{0}^{a/2}\sigma \;dx=\;\Omega \int_{0}^{a/2}\frac{Ga}{2\pi h}\sin\frac{2\pi x}{a}\;dx=\;\Omega \frac{Ga^{2}}{2\pi^{2}h}$$

where Ω was the contact area of adjacent unit cells, on which the stress *σ* acted:

(6) $$\Omega =\frac{3\sqrt{3}}{8}a^{2}$$

The energy *nU* required to shift *n* atoms:

(7) $$nU=\Omega \frac{nGa^{2}}{2\pi^{2}h}=\frac{3nGa^{3}}{4\pi^{2}}$$

As noted above, there were many clusters of adjacent grains in the contact zone, the lattices of which were well matched with each other. These “regions” were separated by zones with increased crystal lattice curvature. Based on this, the shear rate of the upper plane of these regions relative to their lower one, i.e., the rate of an elementary act of intercrystalline sliding, was estimated. In this case, each cluster contained *n* atoms, and its area was equal to *nω*. In the absence of load, the slip activation energy was *E* = *nU*. At stress σ, the activation energy was determined as:

*E* = *nU* ± *σnωa*/2 (8)

where *a*/2 was the distance to the potential barrier between the positions of stable equilibrium. The frequencies of cluster displacements, by the distance *a* in the direction *σ* and in the opposite one, were, respectively:

$$\varsigma_{1}=\varsigma \;e^{-\left(nU-\sigma \;n\omega \;a/2\right)/kT}$$

and

(9) $$\varsigma_{2}=\varsigma \;e^{-\left(nU+\sigma \;n\omega \;a/2\right)/kT}$$

where *ς* was the atomic vibration frequency.

Shear rates in these directions were $v_{1}=\varsigma_{1}a$ and $v_{2}=\varsigma_{2}a$, and the total sliding speed *v* was their sum:

(10) v=v1+v2=a(ς1−ς2)=aςe−nU−σ nω a/2/kT−e−nU+σ nω a/2/kT=aςe−nUkTeσ nω a/2kT−e−σ nω a/2kT=2aςe−nUkTshσ nω a2kT

For small values, *x* sh(*x*) ≈ *x*. Therefore, for a low stress σ, it was possible to write:

(11) v=2aςe−nUkTσ nω a2kT=σ nω a2ςkTe−nUkT

Assuming that the elementary displacement was due to the disordering of atoms inside each cluster, the free energy *F* required for this was zero at the melting point, and at *T* = 0:

*F* = *nL* (12)

where *L* was latent heat of fusion per atom. Based on these equalities, it was assumed that at temperature *T*, the free energy was written as:

*F* = *nL*(1 − *T*/*T_M_*) (13)

where *T_M_* was the melting temperature.

Considering the action of stress σ, a general expression for the shear activation energy *E* was obtained:

*E* = *nF* ± *σnωa*/2 (14)

where a sign before the second term depended on the direction of the stress vector. By analogy with the derivation of Equation (10), it could be written:

(15) v=2aςe−nFkTshσ nω a2kT=2aςe−nL(TM−T)kTTMshσ nω a2kT=2aςenLkTMe−nLkTshσ nω a2kT

At low values of stress σ, this ratio was:

(16) v=σ nω ςa2kTenLkTMe−nLkT

Within the SECA method, the component of the deformation front velocity perpendicular to the boundary of the *i*-th element and its *k*-th neighbor ($v_{ik}^{⊥}$) was calculated as follows:

(17) vik⊥=m0ikσik⊥e−ℑikkBTik

where

(18) $${ℑ}_{ik}=\gamma_{HAGB}\frac{\Delta ({\vec{\theta }}_{i},\;{\vec{\theta }}_{k})}{\theta_{HAGB}}\left(1-\ln\frac{\Delta ({\vec{\theta }}_{i},\;{\vec{\theta }}_{k})}{\theta_{HAGB}}\right)$$

$\sigma_{ik}^{⊥}$ was magnitude of the stress component perpendicular to the element boundary.

Applying (16) to the calculation of the velocity component of the deformation front parallel to the boundary of the *i*-th element and its *k*-th neighbor ($v_{ik}^{\left|\right|}$), the following expression was obtained:

(19) vik||=σik|| nikω ςa2kTikenikLkBTMe−nikLkBTik

The absolute value of the velocity vector was calculated trivially:

(20) $$v_{ik}=\sqrt{{\left(v_{ik}^{⊥}\right)}^{2}+{\left(v_{ik}^{\left|\right|}\right)}^{2}}$$

1. In the case when the misorientation angle of the lattices of neighboring grains was equal to zero: $\Delta ({\vec{\theta }}_{i},\;{\vec{\theta }}_{k})=0$ Relation (18) gave the equality ${ℑ}_{ik}=0$; hence, according to (17),

(21) $${\hat{v}}_{ik}^{⊥}={\left(m_{0}\right)}_{ik}\sigma_{ik}^{⊥}$$

The zero misorientation angle corresponded to the case when the number of atoms in the islands was maximum, i.e., *n_ik_ = n*_max_. Under this condition, Equation (19) was written as:

(22) v^ik||=σik|| nmaxω ςa2kTikenmaxLkBTMe−nmaxLkBTik

The *n*_max_ number could be obtained as follows:

(23) $$n_{\max}=\frac{\Omega_{c}d_{gb}}{V_{a}}$$

where Ω*_c_* was the contact area of adjacent elements, *d_gb_* was the boundary layer thickness, *V_a_* was the volume of a unit cell containing a lattice atom.

2. If the misorientation angle was maximal, i.e., $\Delta ({\vec{\theta }}_{i},\;{\vec{\theta }}_{k})=\theta_{HAGB}$, Formula (17) was:

(24) $${\tilde{v}}_{ik}^{⊥}={\left(m_{0}\right)}_{ik}\sigma_{ik}^{⊥}e^{-\frac{\gamma_{HAGB}}{k_{B}T_{ik}}}$$

At the maximum misorientation angle, the number of atoms in the islands was minimal, i.e., *n*_ik_ = *n*_min_, and Equation (19) was written such as:

(25) v˜ik||=σik|| nminω ςa2kTikenminLkBTMe−nminLkBTik

At any misorientation angle, the amount of energy exchanged between the considered elements was the same, so it was possible to write:

(26) $${\left({\hat{v}}_{ik}^{⊥}\right)}^{2}+{\left({\hat{v}}_{ik}^{\left|\right|}\right)}^{2}={\left({\tilde{v}}_{ik}^{⊥}\right)}^{2}+{\left({\tilde{v}}_{ik}^{\left|\right|}\right)}^{2}={\left(v_{ik}^{⊥}\right)}^{2}+{\left(v_{ik}^{\left|\right|}\right)}^{2}$$

(27) $${\left[{\left(m_{0}\right)}_{ik}\sigma_{ik}^{⊥}\right]}^{2}+{\left[\frac{\sigma_{ik}^{\left|\right|}\;n_{\max}\omega \;\varsigma a^{2}}{kT_{ik}}e^{\frac{n_{\max}L}{k_{B}T_{M}}}e^{-\frac{n_{\max}L}{k_{B}T_{ik}}}\right]}^{2}={\left[{\left(m_{0}\right)}_{ik}\sigma_{ik}^{⊥}e^{-\frac{\gamma_{HAGB}}{k_{B}T_{ik}}}\right]}^{2}+{\left[\frac{\sigma_{ik}^{\left|\right|}\;n_{\min}\omega \varsigma a^{2}}{kT_{ik}}e^{\frac{n_{\min}L}{k_{B}T_{M}}}e^{-\frac{n_{\min}L}{k_{B}T_{ik}}}\right]}^{2}$$

(28) $${\left[{\left(m_{0}\right)}_{ik}\sigma_{ik}^{⊥}\right]}^{2}+{\left[\frac{\sigma_{ik}^{\left|\right|}\;n_{\max}\omega \;\varsigma a^{2}}{kT_{ik}}e^{\frac{n_{\max}L}{k_{B}T_{M}}}e^{-\frac{n_{\max}L}{k_{B}T_{ik}}}\right]}^{2}={\left[{\left(m_{0}\right)}_{ik}\sigma_{ik}^{⊥}e^{-\frac{{ℑ}_{ik}}{k_{B}T_{ik}}}\right]}^{2}+{\left[\frac{\sigma_{ik}^{\left|\right|}\;n_{ik}\omega \;\varsigma a^{2}}{kT_{ik}}e^{\frac{n_{ik}L}{k_{B}T_{M}}}e^{-\frac{n_{ik}L}{k_{B}T_{ik}}}\right]}^{2}$$

In Equation (27) the unknown was *n*_min_, and it was *n_ik_* in (28). Thus, solving Equation (27), the number of atoms in the islands at the maximum misorientation angle was obtained, and the solution of Equation (28) gave the dependence

$$n_{ik}(\Delta ({\vec{\theta }}_{i},\;{\vec{\theta }}_{k}))$$

The $\sigma_{ik}^{⊥}$ and $\sigma_{ik}^{\left|\right|}$ values were computed as follows. Within the SECA algorithm, the $\sigma_{ik}^{⊥}$ value was equal to the difference between the hydrostatic pressures of the element with index *i* (*p_i_*) and its *k*-th neighbor (*p_k_*):

(29) $$\sigma_{ik}^{⊥}=p_{k}^{}-p_{i}^{}$$

At the same time, the element with index *i* and its *k*-th neighbor were characterized by the values of the moments of forces ${\vec{M}}_{i}^{}$ and ${\vec{M}}_{k}^{}$. The magnitude of the moment of force on their boundary ${\vec{M}}_{ik}^{}$ calculated as the average for vectors ${\vec{M}}_{i}^{}$ and ${\vec{M}}_{k}^{}$:

(30) $${\vec{M}}_{ik}^{}=0.5\cdot ({\vec{M}}_{i}^{}+{\vec{M}}_{k}^{})$$

Based on the assumption that the action of the moment ${\vec{M}}_{ik}^{}$ initiated stress ${\vec{\sigma }}_{ik}^{\left|\right|}$ parallel to the boundary of the elements, and the definitions of the moment of force and stress, received the following expression:

(31) $$\left(\Omega_{c}{\vec{\sigma }}_{ik}^{\left|\right|}\right)\times \left(r_{c}{\vec{n}}_{ik}\right)={\vec{n}}_{ik}\times \left({\vec{M}}_{ik}^{}\times {\vec{n}}_{ik}\right)$$

where *r_c_* was the element radius, Ω*_c_* was the contact area of adjacent elements, ${\vec{n}}_{ik}$ was normal to the boundary of elements, the right-hand side of Equation (31) was the projection of the vector ${\vec{M}}_{ik}^{}$ to the boundary of element. Thus, the following expression could be written:

(32) $$\sigma_{ik}^{\left|\right|}=\left|{\vec{\sigma }}_{ik}^{\left|\right|}\right|=\frac{\left|{\vec{M}}_{ik}^{}\times {\vec{n}}_{ik}\right|}{r_{c}\Omega_{c}}$$

Considering (23) and (32), Equation (28) was written as follows:

(33) $${\left[{\left(m_{0}\right)}_{ik}\sigma_{ik}^{⊥}\right]}^{2}+{\left[\frac{\left|{\vec{M}}_{ik}^{}\times {\vec{n}}_{ik}\right|}{r_{c}}\frac{d_{gb}}{V_{a}}\frac{\;\omega \;\varsigma a^{2}}{kT_{ik}}e^{\frac{n_{\max}L}{k_{B}T_{M}}}e^{-\frac{n_{\max}L}{k_{B}T_{ik}}}\right]}^{2}={\left[{\left(m_{0}\right)}_{ik}\sigma_{ik}^{⊥}e^{-\frac{{ℑ}_{ik}}{k_{B}T_{ik}}}\right]}^{2}+{\left[\frac{\sigma_{ik}^{\left|\right|}\;n_{ik}\omega \;\varsigma a^{2}}{kT_{ik}}e^{\frac{n_{ik}L}{k_{B}T_{M}}}e^{-\frac{n_{ik}L}{k_{B}T_{ik}}}\right]}^{2}$$

It should be noted that local moments of forces were a fundamental factor in assessing the effect of the crystal lattice curvature on the intensity of rotational wave flows of defect structures along the grain boundaries [36]. Such flows determined the pattern of material fragmentation, the nucleation of dislocations, disclinations, microcracks, and subsequent material failure. The energy dissipation parameter was used to estimate the fraction of the formed defect structures:

(34) $${\left[\Delta E_{d}\right]}_{\;i}^{\;n}=\frac{k_{diss}G_{i}|\Delta {\vec{\gamma }}_{i}^{n}{|}^{2}\pi r_{c}^{3}}{2}$$

where $\Delta {\vec{\gamma }}_{i}^{n}$ was an increment of the three-dimensional angle of the active element rotation, $G_{i}$ was the shear modulus, $k_{diss}$ was the dissipation coefficient, and $r_{c}^{}$ was the active element radius.

It is known that real mechanical fatigue developed in steels used in pipeline construction during units and even tens of thousands of load cycles. In such a setting, the implementation of the numerical experiments was, first of all, a computationally complex procedure that required significant time and computational resources. Applying the SECA method, the authors implemented a GPU-based procedure that enabled carrying out computational experiments involving hundreds of mechanical load cycles in a foreseeable period of time.

**Figure 12:**
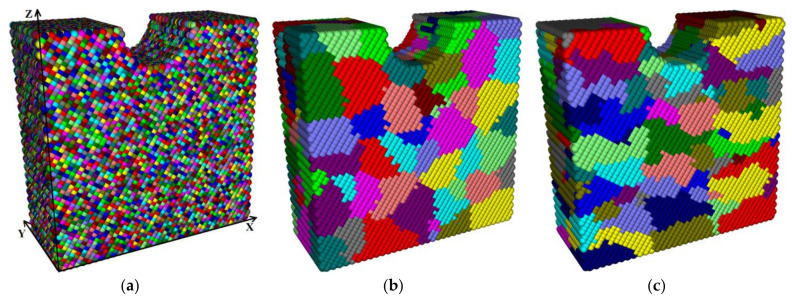
The structures of the model samples with different grain sizes: 2.5 μm (**a**), 20 μm (**b**), and 30 × 30 × 6 μm^3^ (**c**).

Through a series of numerical simulations, the deformation behavior of the samples with different grain sizes and shapes was investigated in order to compare their effect under cyclic loading in terms of resistance to the defect nucleation and subsequent failure. The following presetting was in the computational models. The samples with “coarse” equiaxed grains of ≈20 µm (Figure 12b) corresponded to the as-received 09Mn2Si steel. In the case of the samples cut from the outer layer of the bar after the HR, the grain sizes could be significantly reduced, which corresponded to the structure model shown in Figure 12a. 

In the case of the formation of the longitudinal texture for the samples cut from the bar core, the transverse grain sizes were taken on the order of 6 μm, while the length of the grains increased up to 30 μm (Figure 12c). Each sample (representative mesovolume) had dimensions of 150 × 60 × 150 μm^3^ and a semicircular cylindrical notch in the middle of the upper face with a curvature radius of 25 μm. The size of the cellular automaton element was 2.5 μm, and the time step was 10 ns. The response of the model samples to cyclic loading was studied in detail.

During the numerical experiment with a duration of 1 ms, a cyclic tensile stress was applied from the left and right sides. The duration of one cycle was chosen to be equal to 10 μs. In this case, each cycle consisted of a half-cycle of the load impacting with an effective strain rate of 0.33 s^−1^ for 5 μs and a half-cycle of the unloading, at which the energy inflow was equal to zero. Other faces of the samples were without any loads. In addition, there was no energy exchange with the external environment.

**Figure 13:**
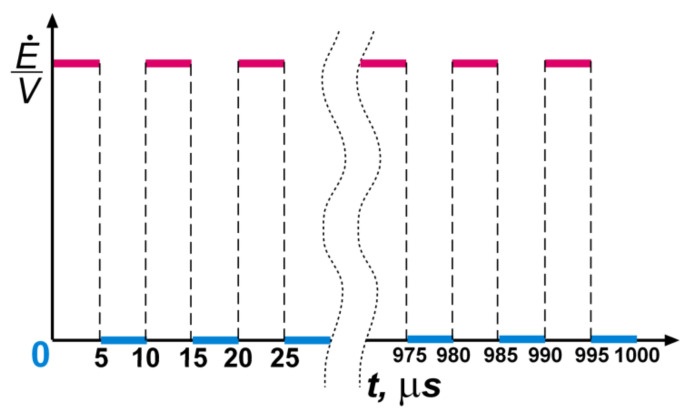
A schematic representation of cyclic loading upon the numerical experiment.

The boundary conditions in the presented numerical method were implemented as follows. A predetermined “portion” of energy Δ*E* was supplied to a plurality of active elements located on the outer loaded sample faces every time step. Then, it propagated deep into the sample as a result of energy exchange. Such “boundary” active elements were marked in the algorithm with special markers, which indicated to the calculated program code that these elements were absolutely elastic and obeyed Hooke’s law. Thus, the amount of energy at the boundaries could always be converted into stresses and strains (or their increment rates). A schematic representation of the loading principle is shown in Figure 13.

**Figure 14:**
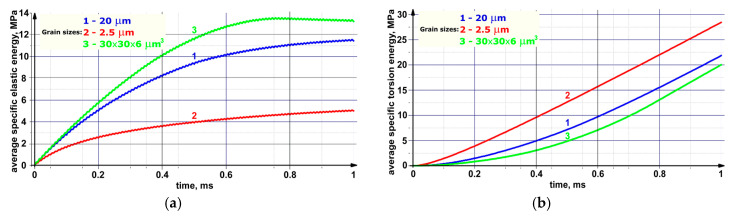
The time dependences of the average values of the specific elastic (**a**) and torsional (**b**) energies for the samples with different grain sizes: 20 μm (blue); 2.5 μm (red), and 30 × 30 × 6 μm^3^ (green).

**Figure 15:**
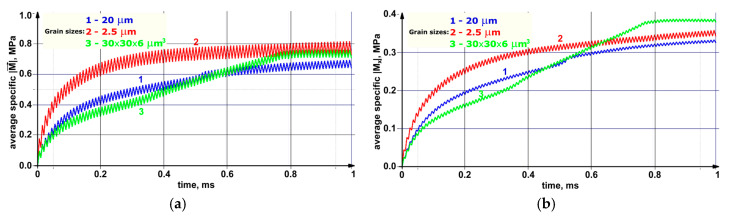

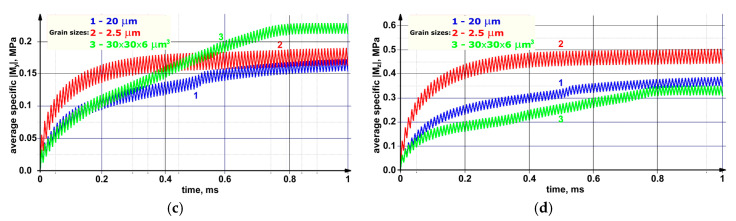
The time dependences of the average values of the specific torque moduli (**a**) and its *X* (**b**), *Y* (**c**), and *Z* (**d**) components for the samples with different grain sizes: 20 μm (blue); 2.5 μm (red); and 30 × 30 × 6 μm^3^ (green).

Figure 14 and Figure 15 show the time dependences of the average volume-specific elastic energy values, the torsional energy, as well as the moduli of the specific moment of force and its *X, Y*, and *Z* components for each of the three considered samples. The graphs presented in Figure 14a show that rate of specific elastic energy gradually decayed with time. This was due to the fact that a part of the elastic energy dissipated as it propagated and was spent on the formation of new defect structures during the local material torsion, which caused a change in its structure. The fraction of the dissipated torsional energy was calculated using Equation (34). These processes were especially intense at the grain boundaries. The increase in the average value of the specific torsional energy in each of the presented samples, as shown in Figure 14b, passed with a gradual “acceleration” due to the involvement of a larger area of the grain boundaries in the elastic energy dissipation process.

As mentioned above, the local torsional energy reached its maximum values at the boundaries of structural elements. In the fine-grained sample (the grain sizes of 2.5 µm), the area of the grain boundaries was the largest and, as a result, the torsion energy level was very high inside (Figure 14b). However, this circumstance intensified the nucleation of new defect structures (dislocations and disclinations) and caused active dissipation of the elastic energy pumped into the sample. This fact explained the low level of the residual elastic energy presented in Figure 14a.

In the sample with grains elongated along the *X* axis, the elastic energy front passed through the smallest area of the grain boundaries in comparison with other ones. Consequently, the elastic energy dissipation was less pronounced in this sample, and the amount of the elastic energy was at its maximum. In this case, there was a “saturation effect” that consisted in the fact that its inflow from the outside slowed down at the high average elastic energy level. As a result, the average elastic energy value stopped increasing at a certain moment (0.75 ms) and then began to decrease due to the dissipation rate enhancing because of the initiation of microrotations at the boundaries of structural elements.

The dynamics of the dependencies shown in Figure 15 confirmed the above statement about the role of the grain boundaries in the development of the dissipation processes. Indeed, the average modulus value of the moment of force, which determined the elastic energy dissipation rate, was maximum in the fine-grained sample during the entire numerical experiment duration. At the same time, a significant increase in the average values of the moduli of the *X* and *Y* components of the moments of forces in the sample with grains elongated along the *X* axis (Figure 15b,c) was very noticeable in the period from 0.3 to 0.8 ms. This phenomenon was mostly due to the aforementioned “saturation effect”, as well as to the fact that the total inflow of the elastic energy was spent on the structure restructuration.

The decrease in the elastic energy level due to the internal structure rearrangement was able to provide improved resistance to the formation of discontinuities in the material under cyclic loading. Based on the obtained results, it could be concluded that the fine-grained structure was the most preferable in this case. At the same time, the structure with elongated grains accumulated a large amount of the elastic energy and demonstrated the ability to reduce its level by increasing the rate of the dissipative processes during the entire loading duration.

**Figure 16:**
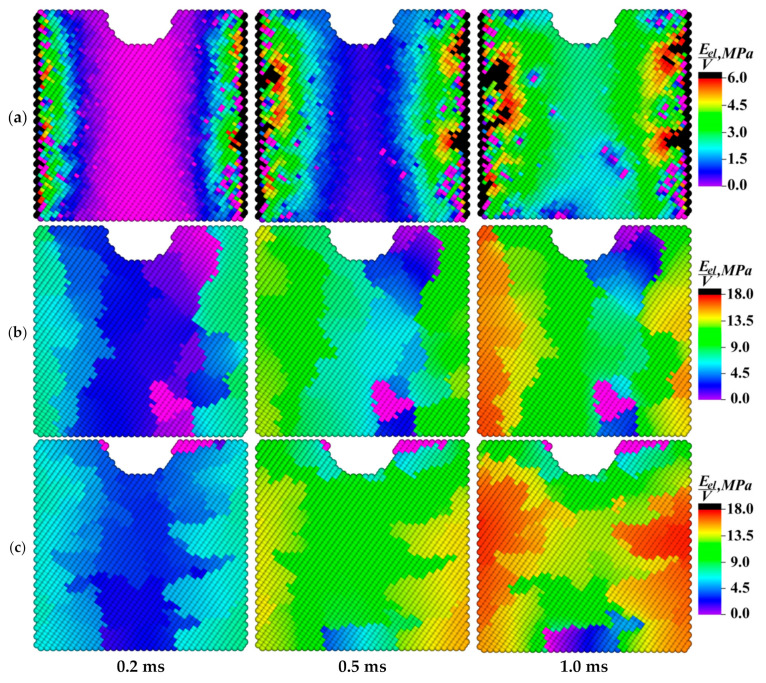
The distribution patterns of the specific elastic energy on the front face of the samples with Table 2. 5 μm (**a**), 20 μm (**b**), and 30 × 30 × 6 μm^3^ (**c**) at different times.

**Figure 17:**
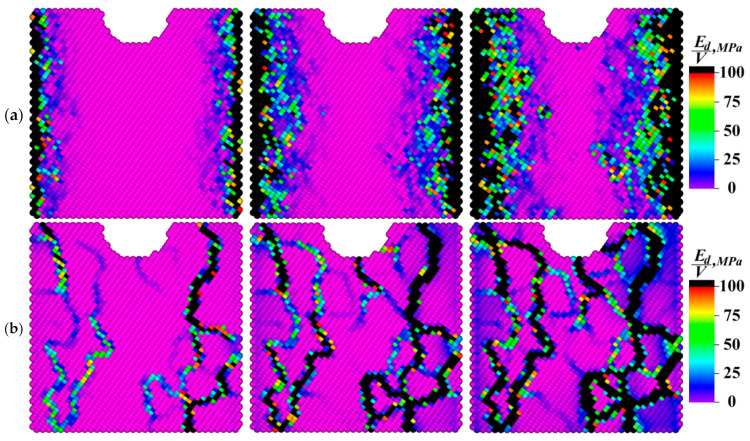

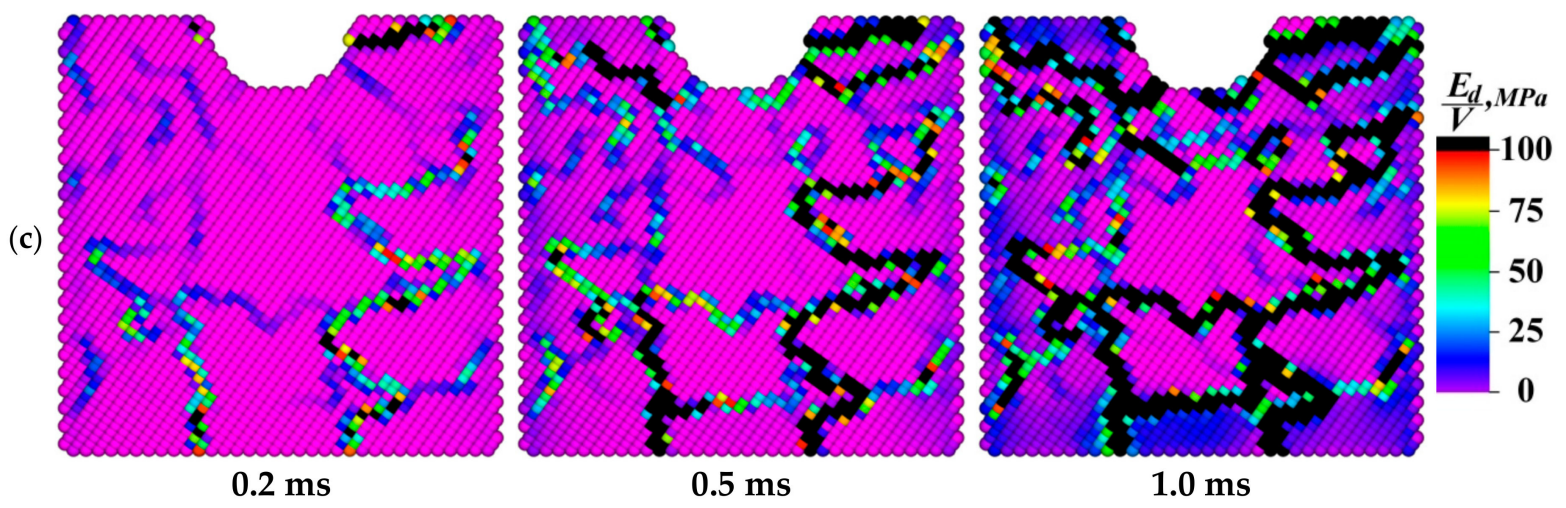
The distribution patterns of the specific torsional energy on the front face of the samples with the grain sizes of 2.5 μm (**a**), 20 μm (**b**), and 30 × 30 × 6 μm^3^ (**c**) at different times.

**Figure 18:**
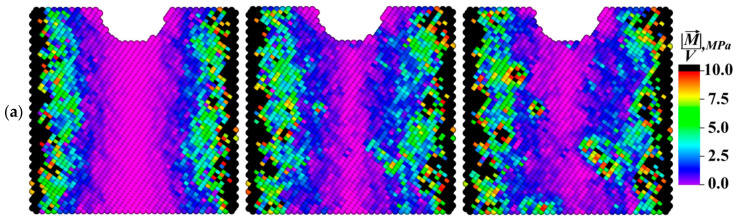

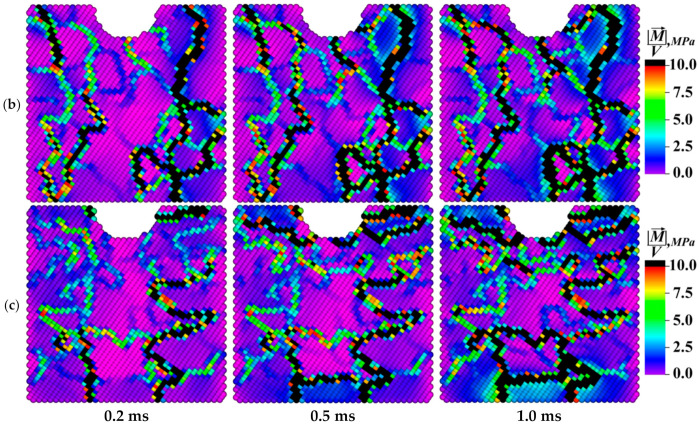
The distribution patterns of the specific modulus of the moment of force on the front face of the samples with the grain sizes of 2.5 μm (**a**); 20 μm (**b**); and 30 × 30 × 6 μm^3^ (**c**) at different times.

**Figure 19:**
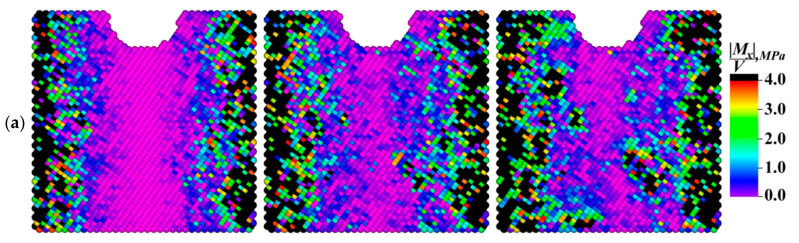

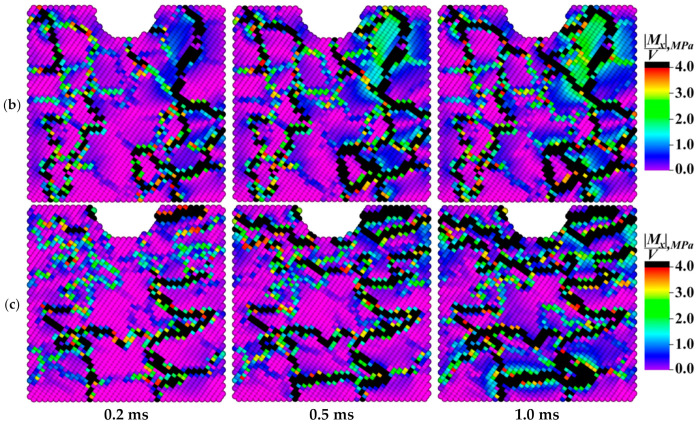
The distribution patterns of the *X* component of the specific modulus of the moment of force on the front face of the samples with the grain sizes of 2. 5 μm (**a**), 20 μm (**b**), and 30 × 30 × 6 μm^3^ (**c**) at different times.

**Figure 20:**
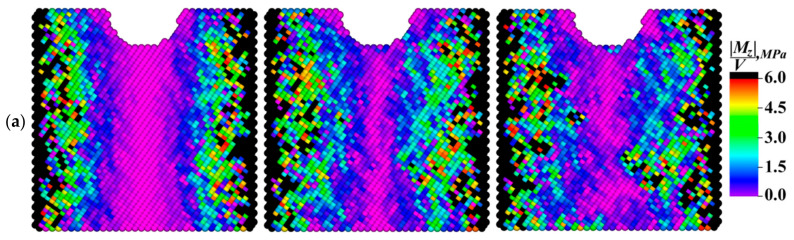

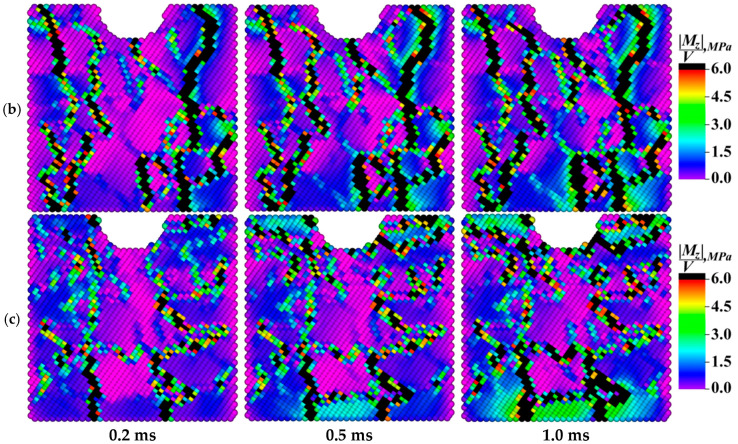
The distribution patterns of the *Z* component of the specific modulus of the moment of force on the front face of the samples with the grain sizes of 2.5 μm (**a**), 20 μm (**b**), and 30 × 30 × 6 μm^3^ (**c**) at different times.

Figure 16, Figure 17, Figure 18, Figure 19 and Figure 20 show value distributions of the specific elastic energy, the specific torsional energy, the moduli of the specific moment of force, and its *X* and *Z* components on the front faces of the samples at different time instants (0.2; 0.5; and 1.0 ms).

The dynamics of the elastic energy distribution patterns presented in Figure 16 testified to the important role of the grain boundaries not only in the effective response of the sample as a whole, but also in the mechanical energy front propagation. The inhomogeneity of the energy distribution was associated with the difference in the misorientation angles at the boundaries. An increase in the misorientation angles of the grains enhanced the boundary resistance to the elastic front propagation, contributing to the intensive formation of defect structures on the one hand and raising the mechanical energy inside the grains on the other. In the fine-grained sample (Figure 16a), the spatial distribution of the elastic energy was the most uniform due to the presence of a large number of branched grain boundaries, which provided a greater number of channels for the propagation of rotational wave energy flows compared to other ones.

In the spatial value distribution patterns of the specific torsional energy (Figure 17), the effect of the accumulation of the rotational strain modes at the grain boundaries was clearly manifested. This phenomenon contributed to the local structure transformation at the boundaries due to the dissipation of the elastic energy fraction, which could result in both an increase in the material ductility and the formation of trans-crystalline cracks. A more uniform distribution of the dissipation energy was observed in the fine-grained sample as in the case of the elastic one.

The spatial value distribution patterns of the moments of forces presented in Figure 18 were similar to the ones for the specific torsional energy by their nature, since the moment of force determined the rate of the material local torsion and the elastic energy dissipation. The maximum mechanical energy gradients were at the grain boundaries, so the moments of forces near the boundaries were also maximum. In the case of the fine-grained structure (Figure 18a), zones of increased values of the specific modulus of the moment of force were gradually formed in the sample core. 

Each contained a conglomerate of several grains. In the samples with the larger size of structural elements, 1–2 grains with a high level of the moment of force were distinguished at the boundaries. This phenomenon could result either in the rotation of the grain as a whole around its axis or in the grain body fragmentation. Such reversals could be mutually compensated within conglomerates of small grains, in contrast to the rotation of larger grains, which could eventually collapse.

The patterns in Figure 19 and Figure 20, in addition to the corresponding graphs in Figure 15, enabled carrying out the qualitative and quantitative comparison of the distribution fields of the values of the *X* and *Z* components of the specific modulus of the moment of force on the front faces of the model samples. The *X* component of the moment of force characterized the rotation around the *X* horizontal axis along which the external mechanical load was transmitted. In turn, the *Z* component characterized the degree of rotation around the vertical axis perpendicular to the external load direction. The qualitative variations between the patterns of the modules of the *X* and *Z* components were almost not noticeable, while there were quantitative ones. 

Due to the very low average value of the *Y* component of the specific modulus in comparison with the other ones, the authors saw no point in discussing its distribution within the framework of the studies. In the case of the fine-grained structure (the grain sizes of 2.5 μm), the average value of the *X* component of the specific modulus of the moment of force was approximately one and a half times less than that of the *Z* component. With an increase in the grain sizes up to 20 μm (for the elongated grains), these values almost leveled off. During the numerical experiment, the average value of the *X* component of the specific modulus in the sample with grains elongated along the *X* and *Y* axes (30 × 30 × 6 μm^3^) increased faster than that of the *Z* component and, as a result, surpassed it by about 10%. This difference enabled revealing the effect of both the grain sizes and their shapes on the sample behavior under the cyclic mechanical loading conditions.

The results of the studies showed that the degree of the elastic energy dissipation in the loaded samples directly depended on the dimensions of the grain boundaries, as well as on the shapes and the sizes of grains. The fine-grained structure showed the highest average torsional energy level among all the investigated samples. This was due to the high density of defects in the form of dislocations and disclinations at the internal boundaries of the material and the decrease in the elastic energy level. This phenomenon was able to provide improved crack resistance under cyclic mechanical stresses. From this point of view, the fine-grained structure was the most effective. During the entire loading, there was a gradual decrease in energy due to the increase in the rate of dissipative processes in the structure with “flat” (elongated) grains, which possessed the highest elastic energy level.

Analysis of the dynamics of changes in the elastic energy value distributions demonstrated the important role of the grain boundaries in the mechanical energy front propagation. It was concluded that the fine-grained structure enabled obtaining the most uniform patterns of the distributions of the elastic and torsional energies.

The effect of the accumulation of the rotational strain modes at the grain boundaries was discovered, which caused the local structure transformation at the boundary zones. In the fine-grained structure, the formation of grain conglomerates was observed, which increased values of the specific modulus of the moment of force. This could be mutually compensated due to the small sizes of grains. At the same time, the coarse-grained structures were characterized by the presence of the small number of grains with the high level of the moment of force at their boundaries. They could result in the trans-crystalline cracking.

It should be noted that severe plastic strains could improve the cyclic durability of materials due to both the microstructure refinement and the grain size reduction. In some cases [50], plastic strains caused the formation of dissipative structures in the materials, especially under dynamic loading. In some papers—for example, [51]—the cyclic crack resistance increased not only at the crack nucleation stage but also at crack propagation one. The authors attributed this fact to a change in the shapes and purity of the grain boundaries.

Before the conclusion, the authors want to highlight the following. According to the review presented in the Introduction section and the obtained results, various mechanisms were identified that were responsible for the change in the stain-strength properties of the materials after the HR processing (from micro to macroscale). All of them, to one degree or another, affected the changes in the 09Mn2Si steel. However, it was rather difficult to quantitatively separate their contribution. As a novelty of these investigations, the authors distinguished three key factors: (a) quantitative identification of the effect of both the sizes and shapes of grains on the change in the mechanical properties; (b) maintaining the effective relaxation mechanisms at the microscale level simultaneously with the improvement of the strength properties; and (c) the results of the analysis of the influence of grain boundaries on strain processes under loading.

## 4. Conclusions

The study of the structure and the mechanical properties of the 09Mn2Si steel after the five-stage HR was carried out. It was revealed that the fine-grained structure had been formed in the surface layer ≈1 mm deep as a result of severe plastic strains. In the lower layers, the “lamellar” structure had been formed, which consisted of thin elongated ferrite grains oriented in the HR direction.

It was shown that the HR provided the gradient increase in microhardness, while its greatest enhancing was in the surface layer 3 mm deep. The preservation of the ductile dislocation mechanisms with simultaneous hardening was associated with the gradient nature of the decrease in the grain sizes and the texture formation in the rolled steel. The formed extensive grain boundaries prevented the plastic strain development, and ferrite grains without significant hardening enabled the accumulation of defects, providing the high relaxation ability, and, accordingly, ductility and toughness.

The five-stage HR resulted in the increase in the steel fatigue life of more than 3.5 times under cyclic tension. The highest values of the number of cycles before failure were obtained for the samples cut from the bar core. The authors found that the main reason for the improved steel fatigue life was the increase in its cyclic strength, the decrease in the grain sizes, and the formation of the “lamellar” structure with sufficient retention of the material ductility.

It was shown that the degree of the elastic energy dissipation in the 09Mn2Si steel samples under loading directly depended on the area of the grain boundaries as well as on the grain shapes. The fine-grained structure possessed the maximum value of the average torsional energy among all the studied samples, which caused the local material structure transformation and the decrease in the elastic energy level. This improved crack resistance under the cyclic mechanical loading.

The numerical experiments showed that the structure with elongated grains accumulated the largest amount of the elastic energy. In this case, the gradual decrease in the energy level was observed due to the increase in the rate of the dissipative processes under loading. Analysis of the dynamics of the elastic energy spatial distribution patterns demonstrated the important role of the grain boundaries in the mechanical energy front propagation. It was shown that the fine-grained structure enabled obtaining the most uniform distribution patterns of both the elastic and torsional energies.

The effect of the accumulation of the rotational strain modes at the grain boundaries was discovered, which caused the local structure transformation at the boundary zones. In the fine-grained structure, the formation of grain conglomerates was observed, which increased the values of the specific modulus of the moment of force. This could be mutually compensated due to the small sizes of grains. At the same time, the coarse-grained structures were characterized by the presence of the small number of grains with the high level of the moments of forces at their boundaries. They could result in trans-crystalline cracking.

It should be noted in the final conclusion, that the authors did not aim to obtain complete quantitative agreement between the experimental and simulation results, since damages accumulated especially in the near-surface layer of the bar upon the HR in addition to the significant refinement of the grain sizes. This could significantly affect the duration of each fatigue failure stage. These studies will be continued in the next investigations by the authors. Nevertheless, the numerical simulation results enable significantly clarifying the specifics of the pre-fracture stage in light of the features of the elastic energy transfer and the dissipation processes through the extremely heterogeneous mesovolume with different structures.

## Figures and Tables

**Table 1:** Chemical composition of 09Mn2Si steel.

C	Si	Mn	Ni	S	P	Cr	N	Cu
≤0.12	0.5–0.8	1.3–1.7	≤0.3	≤0.04	≤0.035	≤0.3	≤0.012	≤0.3

**Table 2:** The results of the static tensile tests.

State	Stress at the Lower Yield Point, MPa	Stress at the Upper Yield Point, MPa	Ultimate Strength, MPa	Elongation, %
As-received	309 ± 20	334 ± 21	467 ± 19	32 ± 5
HR, area 1	621 ± 25	691 ± 27	642 ± 25	13 ± 3
HR, area 2	565 ± 22	622 ± 23	617 ± 21	21 ± 3
HR, area 3	587 ± 24	611 ± 24	659 ± 20	21 ± 4

**Table 3:** The results of the cyclic tension tests.

State	Area	Cycles to Failure *N_f_*, ×10^3^	*N_i_*, ×10^3^	*N_p_*, ×10^3^	*N_i_*/*N_f_*, %
As-received		80 ± 5	24	56	30 ± 4
After the HR	Area 1	200 ± 10 (150% ↑)	20	180	20 ± 2 (33% ↓)
	Area 2	225 ± 12 (181% ↑)	68	157	30 ± 3
	Area 3	305 ± 15 (281% ↑)	137	168	45 ± 5 (50% ↑)
